# Tailored Immersion: Implementing Personalized Components Into Virtual Reality for Veterans With Post-Traumatic Stress Disorder

**DOI:** 10.1192/j.eurpsy.2022.1737

**Published:** 2022-09-01

**Authors:** N. Van Veelen, R. Boonekamp, T. Schoonderwoerd, M. Emmerik, M. Nijdam, B. Bruinsma, E. Geuze, C. Jones, E. Vermetten

**Affiliations:** 1 Leiden University Medical Center, Psychiatry, LEIDEN, Netherlands; 2 ARQ, Centrum ’45, Oegstgeest, Netherlands; 3 Netherlands Organisation for Applied Scientific Research, Soesterberg, Soesterberg, Netherlands; 4 Amsterdam University Medical Center, Psychiatry, Amsterdam, Netherlands; 5 Ministry of Defence, Brain Research And Innovation Centre, Utrecht, Netherlands; 6 UMC Utrecht Brain Center, Department Of Psychiatry, Utrecht, Netherlands; 7 Canadian Forces Health Services, Department Of National Defense, Ottowa, Canada; 8 Ministry of Defense, Military Mental Health Care, Utrecht, Netherlands

**Keywords:** virtual reality, immersion, Veterans, tailored therapy

## Abstract

**Introduction:**

With the application of virtual reality (VR), tailored interventions can be created that mirror the traumatic experiences of veterans with post-traumatic stress disorder (PTSD). Visual elements can be mimicked, and auditory and other senses stimulated. In doing so, the degree of immersion can be adjusted to optimize the therapeutic process. Objectively measuring the sensory immersion is key to keep subjects within their personal window of tolerance. Based on this information the therapist can decide manipulate the sensory stimulation embedded in the treatment.

**Objectives:**

The objectives of this study are to explore the different immersive design aspects of VRET that can be modified to influence the experienced presence in veterans with PTSD, and to discuss possible methods of measuring the emotional response facilitated by immersive design aspects and experienced presence.

**Methods:**

Four design aspects are discussed: system, sensory cues, narrative and challenge. We also report on a user experiment in three veterans that informed on quality and depth of immersion.

**Results:**

Believability of the neutral virtual environment was important for maintaining the veterans’ presence within the VR experience. The immersive design aspects that were personalized and supportive in the narrative of the veteran such as music and self-selected images appeared to have a strong influence on recall and reliving of the traumatic events.

**Conclusions:**

Finally, in order to increase the therapeutic effect in veterans with PTSD, the highlighted design aspects should be recognized and tailored to maximize immersion in virtual reality exposure therapy.

**Disclosure:**

No significant relationships.

